# Experimental Evolution Studies in Φ6 Cystovirus

**DOI:** 10.3390/v16060977

**Published:** 2024-06-18

**Authors:** Sonia Singhal, Akiko K. Balitactac, Aruna G. Nayagam, Parnian Pour Bahrami, Sara Nayeem, Paul E. Turner

**Affiliations:** 1 Department of Biological Sciences, San José State University, San José, CA 95192, USA; akikokaitlin.balitactac@sjsu.edu (A.K.B.); aruna.gomathinayagam@sjsu.edu (A.G.N.); parnian.pourbahrami@sjsu.edu (P.P.B.); sara.nayeem@sjsu.edu (S.N.); 2 Department of Ecology and Evolutionary Biology, Yale University, New Haven, CT 06511, USA; paul.turner@yale.edu; 3 Program in Microbiology, Yale School of Medicine, New Haven, CT 06520, USA; 4 Center for Phage Biology and Therapy, Yale University, New Haven, CT 06511, USA

**Keywords:** Φ6, cystovirus, bacteriophage, experimental evolution, mutational effects, coinfection, genetic exchange, host range, thermostability, genetic robustness

## Abstract

Experimental evolution studies, in which biological populations are evolved in a specific environment over time, can address questions about the nature of spontaneous mutations, responses to selection, and the origins and maintenance of novel traits. Here, we review more than 30 years of experimental evolution studies using the bacteriophage (phage) Φ6 cystovirus. Similar to many lab-studied bacteriophages, Φ6 has a high mutation rate, large population size, fast generation time, and can be genetically engineered or cryogenically frozen, which facilitates its rapid evolution in the laboratory and the subsequent characterization of the effects of its mutations. Moreover, its segmented RNA genome, outer membrane, and capacity for multiple phages to coinfect a single host cell make Φ6 a good non-pathogenic model for investigating the evolution of RNA viruses that infect humans. We describe experiments that used Φ6 to address the fitness effects of spontaneous mutations, the consequences of evolution in the presence of coinfection, the evolution of host ranges, and mechanisms and consequences of the evolution of thermostability. We highlight open areas of inquiry where further experimentation on Φ6 could inform predictions for pathogenic viruses.

## 1. Introduction

**Figure 1:**
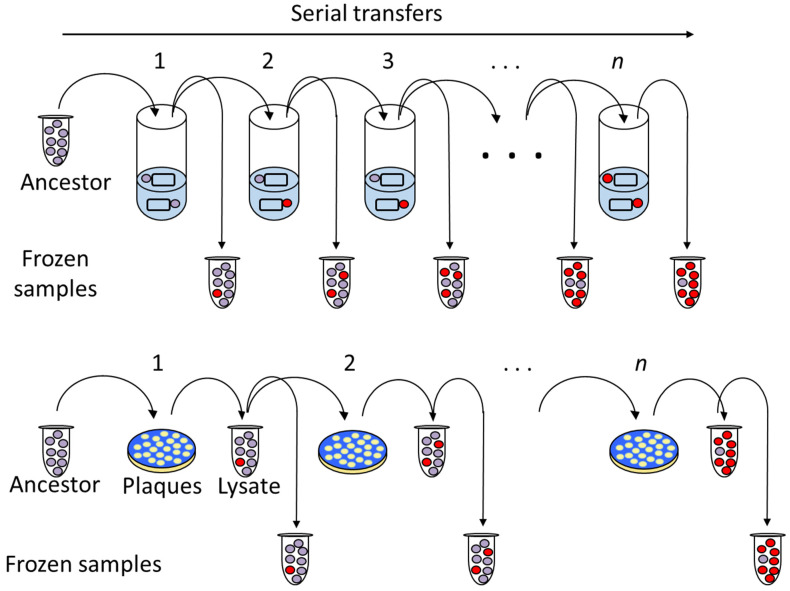
Schematics of evolution experiments using serial transfer of phage populations in the laboratory. Typically, an ancestral clone or population of phages (circles) is allowed to infect bacterial host cells under novel or control environmental conditions. At end of the passage (e.g., after 24 h of incubation), a sample of the phage population is transferred with dilution (e.g., 1:100, creating a bottleneck) into a fresh culture vessel with host cells, and a second sample is stored frozen to permit later analyses. Over time, the phage population is expected to fix mutations (red circles) that increase fitness (survival and/or reproduction) in the novel environment. (**Top**)*:* Phages are serially transferred in liquid cultures containing bacterial host cells (rectangles). (**Bottom**): Phages are grown on lawns of bacterial hosts to form plaques (white circles, indicating zones of lysed bacterial cells). One or more plaques are collected to obtain a cell-free lysate, followed by plating with dilution onto a fresh host lawn.

Experimental evolution studies allow evolutionary processes to be examined in biological populations propagated over time in novel or control environments (Figure 1). Such experiments have proven useful to address a wide variety of topics, including the spontaneous nature of adaptive mutations, the role of population size in determining responses to selection, and the origins and consequences of novel traits. Microbes, which have relatively shorter generation times, larger population sizes, and (sometimes) higher mutation rates than other organisms, have been particularly efficient for studying such “evolution-in-action” [1]. In addition, microbes can be cryogenically frozen and later revived, allowing for direct comparisons between ancestors and their evolved descendants, and their small genome sizes foster affordable recombinant genetic experiments to explore genotype-phenotype mapping. Evolutionary processes that could take years or decades to examine in macro-organisms can be observed in real time in a matter of weeks or months using microbial systems.

In this review, we focus on the rich history of evolutionary studies using the bacteriophage (phage) Φ6. The genome of Φ6 and other cystoviruses is divided into three dsRNA segments (termed L, M, and S for their relative sizes, large, medium, and small) [2,3], making it possible for different Φ6 genotypes to coinfect the same host cell and produce hybrid progeny viruses via the process of reassortment [4]. As an RNA virus, phage Φ6 has an especially high mutation rate (2.7–5.0 × 10^−6^ per nucleotide, [5]) and a short infection cycle (a 70-min [6] to 120-min [7] lysis time). It shares similarities in structure and assembly with eukaryotic Reoviruses [8,9]. These properties have made Φ6 an attractive model for studying various evolutionary questions, such as the effects of mutation accumulation on population fitness, the origins and adaptive maintenance of genetic exchange (sex), the emergence of RNA viruses on novel hosts, and the evolution of virus thermostability. 

## 2. Exploring Fitness Effects of Mutations Using Φ6

### 2.1. Effects of Deleterious and Nearly Neutral Mutations 

Mutations provide the raw material for evolution via natural selection, underlying adaptive traits that affect relative *fitness* (survival and/or reproduction compared to other genetic variants; Box 1). However, aside from mutations that provide such beneficial effects, genetic changes can be deleterious or neutral for fitness. Thus, the frequency and effect distribution of the mutations that occur in a population can heavily impact whether evolutionary processes are adaptive, maladaptive, or neutral over time [10].

Box 1 **Glossary of terms.**

  *Coinfection*: Occurs when two or more virus particles infect the same host cell.

  *Complementation*: A decreased effect of a deleterious mutation, caused by a mutation at a different position or, during viral coinfection, in a different genome.

  *Cost-free*: A change in phenotype or genotype that is not associated with a detectable decrease in fitness.

  *Epistasis*: An interaction between two or more loci that causes a departure from a strictly additive null expectation of their effects. When considering deleterious mutations, a combined effect less deleterious than the null (higher fitness) indicates *positive synergistic epistasis*, and a combined effect more deleterious than the null (lower fitness) indicates *negative synergistic epistasis*.

  *Evolvability*: The capacity for a lineage to undergo greater evolutionary change in future generations.

  *Fitness*: The ability of a genotype to survive and produce offspring, relative to other genotypes in the population.

  *Generalist*: A biological entity that can use multiple different resources, such as a virus that is capable of infecting different host genotypes or species.

  *Genetic drift*: Changes in the allele frequencies in a population across generations, due to random fluctuations.

  *Genetic robustness*: The maintenance of a phenotype despite the introduction of mutations in the genome.

  *Host range*: The number of host genotypes or species that a virus is able to infect.

  *Muller’s ratchet*: The theory proposed by H.J. Muller that predicts that the buildup of deleterious mutations (and hence, fitness decline) is inevitable in an asexual population of a small size.

  *Multiplicity of infection* (MOI): The ratio of virus particles to host cells. At an MOI < 1, cellular infection by individual particles is likely, whereas at an MOI > 1, coinfection occurs with higher probability.

  *Mutational load*: A buildup of mutations in an evolving lineage which can reduce mean fitness over time.

  *Mutation accumulation*: A serial transfer experiment designed to assess the occurrence and frequency of deleterious or nearly neutral mutations empirically, such as asexual populations that experience bottlenecks of a single individual between each transfer.

  *Reassortment*: In multi-partite (segmented) viruses, reassortment is the exchange of genome segments during coinfection, which can result in the production of hybrid progeny.

  *Recombination*: The formation of a new genotype through the breakage and joining of genetic material from multiple (two or more) parental genotypes.

  *Sex*: The exchange of genetic material between distinct parental genomes, resulting in hybrid progeny.

  *Specialist*: A biological entity that uses only a single resource, such as a virus that is confined to infecting one host genotype or species.

  *Thermostability*: The resistance to chemical or physical degradation when exposed to an elevated temperature.

  *Trade-off*: An increase in fitness or performance in one trait at the cost of decreased fitness or performance in another trait.

Natural selection operates less efficiently in populations of small sizes, because random fluctuations in allele frequencies across generations (*genetic drift*; Box 1) cause the inheritance of mutations that are not necessarily beneficial for fitness. The geneticist H.J. Muller posed an idea called ‘*Muller’s ratchet*’ [11,12] (Box 1), which predicted that this process of evolution via genetic drift should be especially problematic in clonal (asexual) populations of small sizes. Similar to a ratchet tool that only moves in one direction, these populations should inevitably accumulate deleterious mutations over time, creating a ‘*mutational load*’ (Box 1) that reduces average fitness. The theory assumes that the rate of reversions and compensatory mutations must be less than that of deleterious mutations. 

**Figure 2:**
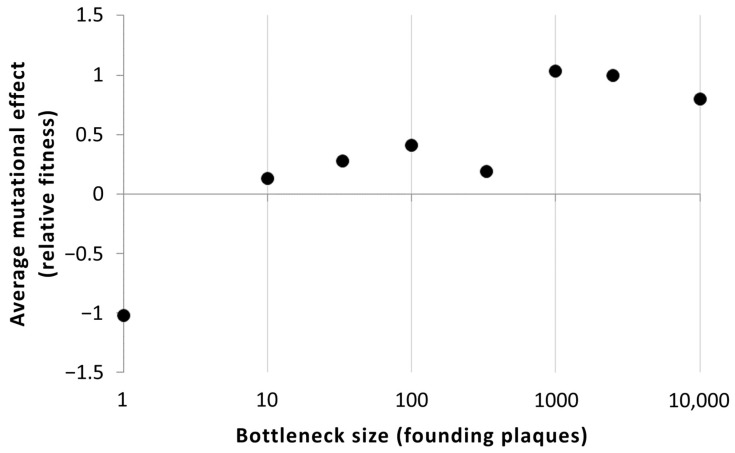
The relationship between bottleneck size during transfers in an evolution experiment (see Figure 1) and the average effect of mutations that reach high frequency in the population after 50–100 transfers. The average mutational effect was calculated as the total difference in fitness between the wild-type ancestor and the final population, divided by the estimated number of mutations. Data are from Table 1 of Burch and Chao 1999 [15].

However, neutral and nearly neutral mutations often go undetected due to absent or minimal fitness effects, and deleterious mutations can be eliminated by selection. This makes it difficult to quantify their effects. To measure the distributions and impacts of small-effect mutations, Chao and colleagues [5,13,14] performed *mutation accumulation* (MA; Box 1) experiments using phage Φ6. During the serial transfer of the virus population onto host lawns (Figure 1, bottom), an extreme bottleneck occurs if a single viral plaque (i.e., the progeny of a single founding genotype) is used to create the lysate that establishes the next generation, because this plaque was formed by a single phage particle. If the plaque is chosen at random, this process allows genetic drift to dominate over natural selection: the chosen plaque may have spontaneous mutations that are either deleterious or have small effects on fitness, and these mutations accumulate over subsequent transfers. Expected decreases in fitness over succeeding generations can be observed by measuring changes in fitness as the MA experiment proceeds (e.g., see Figure 2, bottleneck size = 1). 

Chao (1990) [13] and Burch et al. (2007) [10] performed MA experiments to examine how mutations of deleterious and small effect impacted the fitness of Φ6 populations. Fitness was evaluated either by comparing the growth rate (average particle production) of phages during or after MA relative to that of their founding ancestor [13], or through measurements of plaque size [5,10], known in Φ6 to correlate with particle production measurements [5]. Both studies confirmed fitness decreases over time, consistent with predictions of Muller’s ratchet. By comparing the empirical fitness data to simulations of fitness changes in evolving populations, analyses confirmed that models based solely on the occurrence of deleterious mutations provided a better fit than those based on the occurrence of beneficial mutations, implying that MA lineages fixed harmful mutations [5,10]. Additionally, mutations of small effect were more common than mutations of large effect [10]. 

Burch et al. (2007) [10] also conducted genetic sequencing of evolved phages to connect decreases in fitness with specific underlying mutations. The majority of the mutations detected were single-nucleotide substitutions, located randomly among the three dsRNA genome segments of Φ6. In some MA lines, there were more observed mutations than discrete occurrences of fitness reductions [10], suggesting that some accumulated mutations may have been neutral for fitness.

MA experiments can also be used to evaluate the presence and degree of *epistasis* (interactions between mutations; Box 1). In the absence of epistasis, it is assumed that each additional deleterious mutation will reduce fitness independently from the effects of other genetic changes. However, if deleterious mutations exhibit *positive synergistic epistasis*, then genotypes with additional mutations should experience lesser reductions in fitness (causing higher than expected fitness) as mutations accrue. In contrast, if deleterious mutations exhibit *negative synergistic epistasis*, then genotypes with additional mutations should experience greater losses in fitness (faster declines in fitness than expected).

By tracking changes in fitness across genotypes with differing initial fitness values, Burch and Chao (2004) [5] found that additional mutations in the Φ6 genome had a smaller negative effect on fitness when viruses were already of low (but not high) fitness. This finding suggested that epistasis among deleterious mutations is, on average, positively synergistic in Φ6.

### 2.2. Effects of Adaptive Mutations

In contrast to MA experiments, studies that use very large populations sizes of virus particles for bottlenecks during serial transfers (Figure 1) should favor evolutionary changes due to natural selection rather than genetic drift. Here, beneficial mutations can more easily fix, and adaptation can proceed. Burch and Chao (1999) [15] used a Φ6 clone from a previous MA experiment [13,16] as the founding ancestor of replicate populations propagated under various bottleneck sizes (10, 33, 100, 333, 1000, 2500, and 10,000 individuals). Under the assumption that the distribution of spontaneous beneficial mutations should be dominated by those of small effect, it was predicted that adapting populations must be sufficiently large in numerical size to fix beneficial mutations with strong fitness effects. Results confirmed that populations that were evolved under smaller bottlenecks sequentially fixed mutations with minor fitness effects, while larger bottlenecks permitted more rapid increases in fitness due to the fixation of large-effect mutations (Figure 2, [15]). Thus, the experimental evolution of Φ6 lineages confirmed that the average population size (i.e., the harmonic mean of the bottleneck and the maximum population sizes) influences the opportunity for mutations of differing effect sizes to dictate adaptive change. 

We note that selection experiments can also be used to examine the effects of epistasis on evolution. Burch and Chao (2000) [14] looked at the trajectory of fitness changes in Φ6 populations over time when virus lineages were propagated using either of two different ancestral phage genotypes (viral clones). Each lineage reached different fitness maxima, suggesting that de novo mutations had variable epistatic effects that depended on their occurrences in the distinct genome compositions of the founding ancestors [14].

## 3. Consequences of Coinfection

Evolutionary biologists have long considered the benefits and costs of *sex* (Box 1), broadly defined as the exchange of genetic material between genomes, in populations. On the one hand, a generalized benefit of sex is that it can reduce linkage disequilibrium: sex can recombine multiple beneficial alleles into the same genetic background faster than their expected occurrence via mutation alone, such that adaptation proceeds relatively quickly. Similarly, sex can recombine harmful alleles into common backgrounds, so that selection is more efficient at purging the resulting low-fitness genotypes from an evolving population [17,18,19]. Conversely, sex may break apart beneficial combinations of alleles (e.g., coadapted gene complexes), illustrating a generalized cost of sex [20,21,22]. 

**Figure 3:**
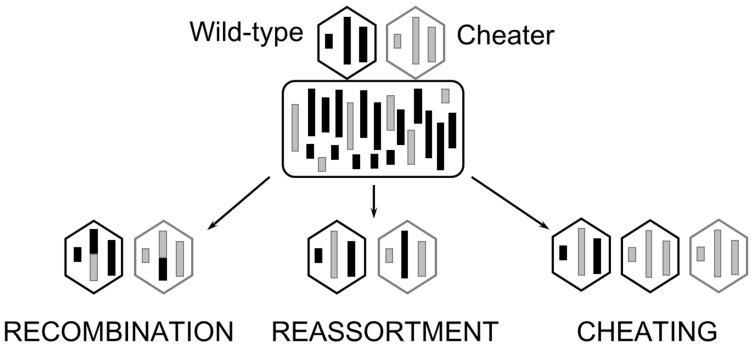
Possible consequences of coinfection in Φ6. Φ6 (the hexagons) has three genetic segments (vertical bars), named Small, Medium, and Large for their relative lengths. When two or more Φ6 phage particles infect a bacterial cell (the rounded rectangle), they are able to exchange genetic information. In the case of recombination, crossover during replication results in segments that contain genetic information from each phage parent. In the case of reassortment, the new phage packages an entire, genetically different segment. Phage particles may also ‘cheat’ by preferentially packaging one or more of their own segments into the capsid of a genetically distinct phage.

Viral sex cannot occur unless multiple, genetically distinct particles coinfect the same host cell. Opportunities for viral sex should thus be determined by the probabilities for *coinfection* (Box 1), which can be manipulated experimentally by controlling the *multiplicity of infection* (MOI), or the ratio of phage particles to bacterial cells. Assuming that a Poisson process determines the extent of coinfection at any given MOI, at relatively low MOI (<<1.0), it is expected that most infections will occur through a single phage entering a host cell on its own, which favors asexuality (clonality). By contrast, at relatively high MOI (>>1.0), the prediction is that the vast majority of infections will involve multiple (2 or more) phages entering one bacterium, which fosters sexuality [23]. The tripartite genome of Φ6 (three dsRNA segments per phage particle) can permit the formation of hybrid progeny viruses containing segments from different coinfecting virus ‘parents’ [24] (Figure 3), a process known as *reassortment* (Box 1). (*Recombination*, the breaking and joining of homologous segments, is either nonexistent in natural Φ6 populations or occurs at rates too low to measure [25,26,27].) Early experiments in Φ6 found that reassortment alone allowed mutated segments to be combined into low-fitness hybrids that were then eliminated by selection, reversing Muller’s ratchet and supporting an advantage of sex [13,16,19].

### 3.1. Constraints on Adaptation during Coinfection

The ability for sex to combine independent beneficial or deleterious mutations for rapid improvement in population fitness suggested that sex may alter the rate at which Φ6 lineages adaptively improve growth on their host bacteria. Turner and Chao (1998) [23] leveraged the ability to manipulate the MOI of Φ6 populations to control the frequency of coinfection, and hence whether sex is common versus infrequent during the viruses’ adaptive process. Three lineages of Φ6 were transferred for 250 virus generations at an MOI of 0.002 (i.e., from 4 × 10^6^ phages/mL to 2 × 10^9^ bacteria/mL), whereas three other lineages experienced an MOI of 5 (i.e., from 1 × 10^10^ phages/mL to 2 × 10^9^ bacteria/mL). Phages were allowed to adsorb to their bacterial host in liquid at the treatment MOI (0.002 or 5), and were then plated on lawns of uninfected bacteria. To control phage population sizes, different numbers of plaques were collected (Figure 1, bottom) for the next round of adsorption: 500 plaques (each resulting from the progeny of one adsorbed phage) were collected from the low-MOI populations, and 100 plaques (each resulting from the progeny of five adsorbed phages) were collected from the high-MOI populations. The fitness of the evolved phages was measured at different time points in evolution by comparing the growth rate (average particle production) of the test phages relative to that of the common ancestor phage. 

If genetic variation generated through sex (reassortment during coinfection) was beneficial for adaptation, the high-MOI populations would show relatively greater fitness than their clonal counterparts by the end of the experiment. However, this hypothesis was not generally supported. In the low-MOI populations, the relative fitness in the selected (MOI = 0.002) and unselected (MOI = 5) environments did increase monotonically over evolutionary time. However, the high-MOI populations increased in fitness in their selected (MOI = 5) environment, but performed poorly in the unselected (MOI = 0.002) environment. This context-dependent result indicated that the Φ6 lineages evolved at a high MOI suffered a performance trade-off. That is, the fitness of these virus populations evolved under frequent coinfection relied on the continued ability to experience coinfection, otherwise they showed poor performance in the low-MOI (clonal) environment [23]. 

This result was later explained by the realization that a high MOI permitted not only the opportunity for reassortment, but also caused selection to differ between the two treatments [28]. In the low-MOI populations, phages experienced selection to better exploit the host cells to produce progeny, whereas in the high-MOI populations, frequent coinfection caused phages to experience intracellular competition as well. Further study of a representative phage genotype that had evolved at a high MOI, ΦH2, revealed that it had higher fitness relative to the wild-type ancestor virus in the presence of coinfection, but lower fitness when infecting cells on its own. This result was consistent with the famous Prisoner’s Dilemma outcome in evolutionary game theory, which explains how cheating can be positively selected in biological systems despite the evolution of *lower* fitness when cheaters take over the population [28]. Measurements of viral traits for ΦH2 confirmed that, when infecting cells on its own, the genotype was less productive (had a smaller burst size) than the wild-type ancestor, indicating that when coinfecting host cells, ΦH2 ‘cheats’ by somehow gaining a productivity advantage over non-cheater viruses [29,30]. 

**Figure 4:**
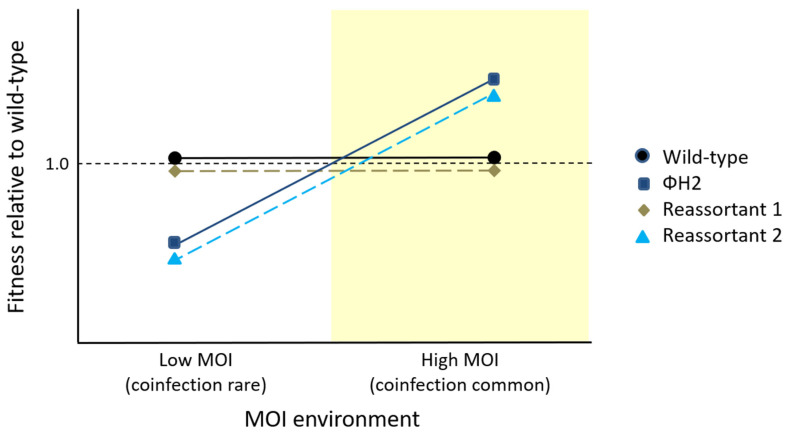
Hypothesized fitnesses of wild-type Φ6, ΦH2 (cheater), and hybrid reassortants containing one genomic segment of ΦH2 in the wild-type background when infecting host cells. ΦH2 performs worse than the wild-type Φ6 at a low MOI, but better than the wild-type Φ6 in a mixed, high-MOI infection. Reassortant 1 shows no evidence that its effect on host bacterial growth differs from that of the wild-type (i.e., the introduced genomic segment does not contain mutation(s) from ΦH2 for poor productivity at a low MOI or high productivity at a high MOI). By contrast, Reassortant 2 recapitulates the poor productivity of ΦH2 on host cells at a low MOI and its high productivity at a high MOI, suggesting that its introduced segment likely contains at least one mutation associated with these phenotypes in ΦH2.

Turner and Chao (2003) [30] offered possible explanations for how cheating could function mechanistically in ΦH2, based on observations of evolved cheaters in other virus systems [31,32,33], but the details of the proposed mechanism have not been confirmed to date. One possibility is that ΦH2 is relatively inefficient in making capsids when infecting cells on its own [29], but is biased in favor of placing its own genome into all procapsids when coinfecting a cell with the wild-type or other non-cheater viruses. If true, the underlying basis for biased procapsid entry must be due to mutation(s) that distinguish the ΦH2 cheater from its wild-type ancestor. Support for this idea would be the observation that one or more segments in the cheater virus are necessary and sufficient to reduce ΦH2 productivity during clonal infection at a low MOI, and to increase ΦH2 productivity in a mixed, high-MOI infection. This can be examined by introducing each evolved segment of the cheater virus into the wild-type genomic background and testing whether any of the resulting reassortant hybrids shows a growth disadvantage in the low-MOI environment, and a growth advantage at a high MOI (Figure 4). Interestingly, preliminary data are consistent with the first hypothesis, and suggest that the evolved M segment of the ΦH2 cheater is alone capable of recapitulating the poor productivity of this virus at a low MOI. The hypothesis of biased procapsid entry is also consistent with the earlier use of a marker on the M segment to measure the fitness of the ΦH2 cheater relative to the wild-type Φ6 in competition assays [23,28,30], suggesting that the allele(s) responsible for cheating could reside solely on the M segment. Further confirmation, however, must come from experiments demonstrating that the evolved M segment is also necessary and sufficient to explain the fitness advantage of the ΦH2 cheater at a high MOI, and that one or more mutations on this segment can drive the cheater’s preferential procapsid entry during coinfection. 

### 3.2. Constraints on Genetic Diversity due to Coinfection

Frequent versus infrequent coinfection should also be consequential for average genetic diversity in phage populations. Because sex allows for the creation of genetic variation beyond typical changes (point mutations, insertions, deletions), virus populations evolved at a high MOI should be more genetically diverse than lineages evolved under clonality. After 300 generations of experimental evolution at a high or a low MOI, ten clones (plaques) from treatment populations of Φ6 were chosen at random and partially sequenced [34]. Contrary to expectations, the low-MOI (clonal) populations showed higher average genetic diversity (13 mutations) compared to their high-MOI (sexual) counterparts (4 mutations). This difference was largely due to greater numbers of mutations in the low-MOI populations in the 5’ untranslated regions of the S segment. Dennehy et al. (2013) [34] also observed eight different mutations in the P5 lysis protein, five of which were synonymous substitutions. Two of the synonymous mutations were beneficial for fitness measured through competition assays. One of the beneficial mutations in P5 (a change from *g* to *u* at nucleotide gene position 591) was found to occur in both the low-MOI and high-MOI treatment populations, suggesting it provides a generalized advantage for virus growth, whereas the other beneficial P5 mutation (a change from *c* to *u* at nucleotide gene position 507) was observed only in a low-MOI population [34]. 

Based on these observations, the frequent opportunity for reassortment in phage Φ6 populations cultured at a high MOI seems to *reduce* the average genetic variation, despite contrary expectations. The explanation could be that dsRNA segments that contain deleterious mutations are maintained in virus populations experiencing frequent coinfection, because their deleterious effects can be masked (*complemented*) in trans by higher fitness phages during intracellular coinfection [23,28,35] (Box 1). This effect would cause weaker selection for removal of deleterious mutations from the virus genome [35]. Furthermore, frequent coinfection could foster exchange of entire segments (reassortment) at high enough rates that variability is homogenized in a phage population. In contrast, a low-MOI environment can allow clonal interference, where independent viral mutants compete with each other to fix in the phage population. Mutants of similar or equal fitnesses may persist for prolonged periods, increasing genetic diversity in asexual populations [34]. Effects of clonal interference may also explain why deleterious mutations in Φ6 were observed to be purged faster in the *absence* of coinfection [35].

The rates of fitness improvement and levels of standing genetic variation observed in Φ6 experimental evolution studies tend not to support expectations for the evolutionary benefits of sex. But these expectations assume that the evolutionary origin and/or adaptive maintenance of viral sex in phage Φ6 is beneficial, and arose for the purpose of promoting variation. The segmentation of the dsRNA genome in Φ6 and other viruses may have evolved for other reasons, such as the efficient packaging of the RNA genome into capsids during intracellular replication [4,36]. 

## 4. Host Range Evolution

The *host range* (Box 1) of a virus is the number of hosts (genotypes or species) that it can infect to produce progeny particles. Although the host range should ideally include all of the native host(s) that the wild-type virus typically infects, it is difficult or impossible to know the true host range for a virus in its natural environment. Thus, the study of virus host ranges in the laboratory often explores how mutational changes can either expand (to include additional novel hosts) or reduce (eliminate the ability to infect one or more original hosts) the number of susceptible hosts. *Generalist* viruses, which have a relatively broader host range, seem to be more likely to emerge successfully on new hosts, whereas *specialist* viruses with relatively narrower host ranges may be constrained in emergence [37,38,39,40,41] (Box 1). Therefore, studies of host-range evolution in viruses are fundamentally important for understanding the mechanisms that make viruses more or less likely to emerge on new hosts in the future.

Phage Φ6 was originally isolated from bean straw infested with *Pseudomonas syringae* pathovar *phaseolicola* bacteria (later reclassified as *P. savastanoi* pv. *phaseolicola* [42,43]), which acts as the typical host in laboratory studies. Wild-type Φ6 also has the ability to infect several related bacterial species: *P. syringae* pvs. *persicae* and *tagetis*, and *P. savastanoi* pv. *savastanoi* [44]. Furthermore, Φ6 can easily gain single-nucleotide mutations that expand its host range to infect novel hosts, including *P. savastanoi* pv. *glycinea* [45], *P. syringae* pv. *tomato* [44,46,47] and pv. *atrofaciens* [6,44,46], and *P. pseudoalcaligenes* East River isolate A (ERA) [44,46,48]. The ability for Φ6 to infect various hosts natively and to emerge on others via single-step mutations has made it an attractive model for studying viral emergence and the evolutionary genetics of virus specialism and generalism.

### 4.1. First-Step Mutations Fostering Emergence on Novel Hosts

Host-range mutants of Φ6 with broadened infectivity on novel host(s) often show fitness decreases on the original host. Φ6 mutants that infect ERA [49,50], *P. glycinea* [45], *P. tomato* [49], or *P. atrofaciens* [46,49] typically have lower fitnesses than wild-type Φ6 on the typical lab host, *P. phaseolicola*. These studies suggest possible *trade-offs* (Box 1), where expanded host-range genotypes that infect a novel host may pay a performance cost of suboptimal or lack of growth on the native host, at least initially. However, these outcomes also depend on the particular host-range mutations experienced by Φ6 [45,50]. For *P. glycinea* host-range mutants, there is a positive correlation between the fitness on novel (*P. glycinea*) and laboratory (*P. phaseolicola*) hosts [45], whereas host-range mutants isolated on ERA bacteria do not show this general pattern [50].

**Figure 5:**
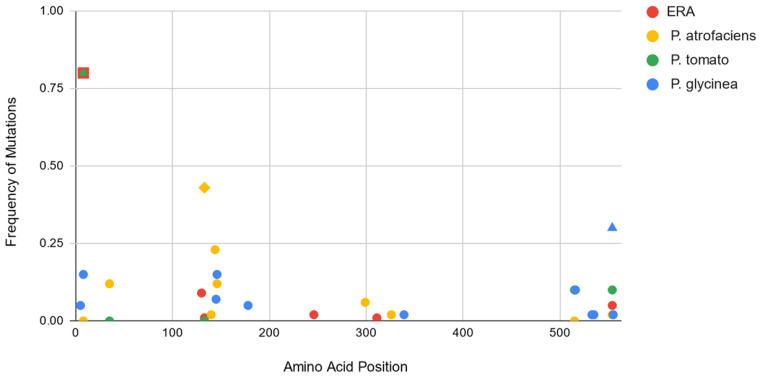
The frequency of host-range mutations across the Φ6 P3 spike protein. The mutants were isolated on the specific host (colored circles), and the *p3* gene was sequenced to identify mutations. The number of mutations at each P3 residue isolated on a particular host was divided by the total number of P3 mutants that were isolated from that host to obtain the frequency of mutations. Residues discussed in the text are marked with a square (residue 8), diamond (residue 133), and triangle (residue 554). Data are from [44,45,46,50].

The mutations that allow Φ6 to emerge on a novel host tend to be nonsynonymous substitutions located in the gene that encodes the P3 spike protein, which interacts with the host-cell receptor. Across four studies [44,45,46,50], 142 different amino acid substitutions have been inferred to underlie changes in the host range of Φ6. In Figure 5, we show the frequency with which known host-range mutations in Φ6 have been isolated on novel hosts, and indicate their positions within the *p3* gene that encodes the spike protein. We note that a few of these mutations, despite being isolated on one host, facilitate the infection of additional novel hosts. For example, amino acid mutations D35A, initially isolated on *P. atrofaciens*, and G515S, isolated on *P. tomato*, also allow for the infection of ERA; and S246T, isolated on ERA, also allows for the infection of *P. atrofaciens* [44].

Figure 5 shows that changes at the *p3* locus relate to differing abilities for these mutants to infect certain host bacteria. Mutations at residue 8 (the square in Figure 5) occur at a higher frequency when Φ6 is challenged to infect ERA and *P. tomato* bacteria, while host shifts onto *P. glycinea* favor mutations at residue 554 (the triangle in Figure 5), and host shifts associated with *P. atrofaciens* emergence are favored by mutations at residue 133 (the diamond in Figure 5). According to a study that examined these mutations in light of the structure of the P3 protein, all three aforementioned amino acid changes were predicted to be topologically close together [50]. This region of the protein may have a particular functional role in the attachment of Φ6 to host cells, with different amino acid replacements responsible for specific changes in the host range. 

Interestingly, prior evolution of Φ6 on native *P. phaseolicola* bacteria also seems to produce genetic and phenotypic changes that affect the potential for Φ6 to productively infect other hosts. For example, Φ6 populations that were previously evolved on *P. phaseolicola* at a low MOI demonstrated higher rank order fitness when challenged to grow on *P. tagetis* and *P. savastanoi* host bacteria, compared to their counterpart populations that experienced evolution at a high MOI [7]. The relatively larger burst sizes of viruses evolved at a low MOI [29] may have increased the likelihood that some progeny viruses would establish infections on these novel hosts [7]. Differing selective pressures associated with strong versus weak population growth of evolved viruses in one host environment may thus also be consequential for the probability for successful viral emergence on alternative hosts.

### 4.2. Evolutionary Adaptation on Novel Hosts

In nature, viruses are expected to shift onto novel hosts in steps. Initially, an ancestral virus can only infect the host(s) constituting its original niche. The first stage of emergence requires that one or more spontaneous mutations occur that broaden the ability of some variants in the virus population to infect both the native and additional host(s). At the next stage, selection to improve infection of the novel host can lead to virus adaptation (increased fitness) [45]. Single-nucleotide host-shift mutations in the first stage must arise in a limited time to permit the initial infection of a novel host, but the expectation is that sustained transmission on the new host may not be possible. Thus, the second stage, involving further adaptive evolution on the novel host, may result in viruses that become specialists on the new host and can no longer infect the original (native) host(s). However, this simple example does not include the many possibilities where emerging viruses might encounter complex environments, especially ones that permit interactions with both native and novel hosts through time. For example, virus evolution may occur on hosts that vary across space (heterogeneous host environments) or through time (alternating host encounters). Here, selection should favor the evolution of generalist viruses that can infect multiple hosts. Several evolutionary studies on phage Φ6 have tested scenarios where virus populations must maintain generalism over long periods of time on native and novel hosts, or specialize on novel hosts.

When serially transferred in environments with a heterogenous mixture of native and novel hosts, Φ6 populations were observed both to evolve generalism and to maintain generalist traits over time. Evolved generalists grown with *P. atrofaciens* or ERA bacteria hosts rarely demonstrated a fitness cost between performance on novel and native hosts [6,51,52], and these lineages could even outcompete evolved specialist populations if the relative proportions of novel hosts in the environment were sufficiently abundant [6,51]. Evolved generalists also continued to bind (adsorb) more rapidly to the cells of the native host than to those of the novel host [51]. These results indicate that evolved generalism in phage Φ6 can be ‘*cost-free*’ (Box 1), in terms of avoiding strong fitness trade-offs across host environments as emergence proceeds. Evolution in heterogeneous host environments may maintain selection for the infection of both hosts, with the native host as a priority for selection in generalist viruses and the novel host as an alternative resource when the native host cells are depleted.

Φ6 selection experiments also demonstrate that generalism can be maintained in alternating (temporally heterogeneous) host environments. Zhao and Duffy (2019) [46] challenged generalist populations and specialist populations to evolve for 30 serial transfers in experimental treatments containing either *P. phaseolicola* bacteria alone, or with alternating infection of *P. phaseolicola* and novel host bacteria at each transfer. When a specialist or a generalist population was transferred solely on *P. phaseolicola*, there was no significant difference in observed fitness gains through time. However, when these populations were serially transferred on alternating hosts, fitness on *P. phaseolicola* either decreased (performance trade-off) or showed no significant difference from the ancestor’s performance. Populations evolved on alternating hosts always improved in fitness on the novel host used in selection, but fitness on another (unselected) novel species in the host range either remained the same or decreased over time [46].

However, heterogeneous host conditions that facilitate generalism may reduce genetic diversity, because mutations that are beneficial or neutral in one host environment may be deleterious in another [1,40,44,53]. In support of this hypothesis, Zhao and Duffy (2019) [46] found that, regardless of whether the founding phage was a specialist or a generalist, populations transferred solely on *P. phaseolicola* exhibited a higher normalized number of single-nucleotide polymorphisms (SNPs) than populations that experienced infection of alternating host types. This suggests that a constant or native host environment produces a greater diversity of genotypic variants than a fluctuating host environment.

Evolution in simpler environments consisting of a single novel host, on the other hand, should be more likely to result in trade-offs suffered by viruses on the native host. In one study, Φ6 generalists that were serially transferred solely on ERA host bacteria experienced reduced attachment rates on the original *P. phaseolicola* host [52], despite their continued ability to infect both hosts. A separate study observed that adaptation to a single host led to mutational changes which *reduced* the host range of Φ6, or even resulted in its complete specialization on the new host [49]. Here, when a generalist genotype with an E8G amino acid substitution in the P3 spike protein was evolved for 30 serial transfers on ERA host bacteria, the phage populations accumulated amino acid changes in P3 that narrowed the host range from six hosts *(P. phaseolicola*, *P. persicae*, *P. savastanoi*, *P. tagetis*, *P. tomato*, and ERA) to either five hosts (amino acid substitution A31T caused an inability to infect *P. tomato*) or just one host (mutants with amino acid substitution G247A could only infect ERA bacteria) [49]. Individual point mutations can thus explain narrow host-range traits in Φ6, including extreme specialization for an evolved lineage that could no longer infect the native host and could only infect ERA. 

The ecological dynamics for emerging virus persistence and expected evolution potential on novel hosts can be examined in short-term studies that show whether viruses can avoid extinction when challenged to thrive on native and novel hosts. Using phage Φ6 populations, these experiments demonstrated that the probability of emergence should depend on the size of the virus population. Dennehy et al. (2006) [48] cultured Φ6 populations in environments that alternated daily between infection of novel ERA hosts and native *P. phaseolicola* hosts. The authors found that lineages had higher fitnesses at large population sizes, and lower fitnesses when population sizes were small. Across the population sizes examined, the mean fitness was observed to be higher for viruses experiencing alternating host treatments than for those propagated on the novel ERA bacteria alone. This study showed that the persistence of emerging viruses on a novel host should be more likely at relatively large population sizes or with a native source population, where the virus is instead expected to go extinct on the novel host alone.

## 5. Thermostability Evolution

### 5.1. The Direct Evolution of Thermostability

The evolution of viruses on new hosts receives abundant attention in virology owing to the importance of disease emergence. But viruses also experience abiotic changes in their natural environments, and an inability to withstand such perturbations can prove costly to infectivity. Several evolution studies in Φ6 have explored how viruses can resist heat shock, or evolve greater *thermostability* (Box 1), in environments that manipulate the frequency of an elevated-heat exposure [54,55,56]. Φ6 is typically cultured in the laboratory at a standard temperature of 25 °C. However, when exposed to 5-min heat shocks at extreme temperatures such as 45–50 °C, the survival of the wild-type virus population decreases substantially. Periodic exposure to heat shocks, in between serial transfers that permit growth on bacteria at 25 °C (Figure 1, top), are shown to increase the average survival of viruses at the elevated temperature. Point mutations that improve Φ6 survival in the face of thermal stress commonly map to the P5 lysis protein [55,56,57] and the P8 outer shell protein [56]. The P5 and P8 proteins are located on the outer surface of the virus particle’s membrane, and are necessary for attachment to and initial infection of host cells [58,59], making them vital structures for degradation avoidance under thermal stress. The V207F amino acid substitution in P5 is especially commonly observed in these studies [55,56,57]. Crystal structure comparisons of the wild-type P5 protein and a P5 protein with the V207F substitution revealed that phenylalanine fills a hydrophobic pocket in the P5 protein. This mutation may thus enhance the thermostability of P5 by increasing the molecular van der Waals interactions between amino acids, improving the survival of Φ6 under heat stress [55]. 

However, a higher thermostability of proteins, particularly enzymes, may not be generally beneficial for fitness. Many enzymes undergo conformational changes to catalyze reactions, but increasing molecular forces within the protein for greater thermostability may result in ‘stiffness’ that hinders catalytic function [60,61]. In Φ6, certain thermostable mutations demonstrate this expected fitness trade-off. For example, wild-type-derived mutants with V207F exhibit higher thermostability, but a decreased viral growth rate at 25 °C [55], suggesting a trade-off between thermostability and growth. However, the effect in Φ6 may also be genotype-dependent (V207F in a non-wild-type genetic background increased both survival to heat shock and growth rates at 25 °C [56]). Comparisons of the growth rate and thermostability of 10 single-nucleotide P5 and P8 mutants of Φ6 also did not support a generalized trade-off between these traits [56], indicating that genetic architecture matters for their interplay. 

### 5.2. Thermostability and Genetic Robustness

Interestingly, selection in different biotic environments also influences the ability of Φ6 to survive under abiotic thermal stress. Clones from Φ6 populations evolved at a low versus a high MOI for 300 generations (an extension of the coinfection-manipulated experiments performed by Turner and Chao, 1998 [23]) showed both equivalent fitnesses at a low MOI and equivalent survival to 45 °C heat-shock exposure. However, when these populations were further evolved for 10 serial transfers in the presence of periodic heat shocks, the increase in survival at 45 °C was greater for the viruses evolved previously at a low MOI than for those evolved previously at a high MOI [54]. When permitting these viruses to evolve under periodic heat shocks when the populations were initially genetically diverse, the viruses previously evolved at a low MOI also adapted faster in their thermotolerance to 45 °C than did their high-MOI counterparts [57].

These results suggest that viruses evolved at a low MOI may be advantaged in accessing beneficial thermostabilizing mutations. For example, if the low-MOI-evolved viruses had a higher spontaneous mutation rate than the high-MOI-evolved viruses, they could be capable of gaining adaptive thermostability more rapidly because of the higher input of mutations. However, tests of the frequency of specific beneficial mutations, either a host-range mutation or a mutation that confers resistance to a chemical (butylated hydroxytoluene) that cleaves the P3 protein, revealed no measurable differences in the spontaneous mutation rate between viruses evolved previously at a low versus high MOI [54,62]. Alternatively, viruses evolved at a low MOI may have possessed one or more mutations that predisposed them to the positive effects of input thermostabilizing mutations. In support of this hypothesis, low-MOI-evolved viruses had greater mean survival than high-MOI-evolved viruses at moderately high temperatures of 40–42 °C [63]. Sequencing of the resulting heat-shocked populations revealed that the P5 mutation V207F, described above, was correlated with these increases in survival [57]. 

A third hypothesis, perhaps more intriguing, is that viruses evolved at a low MOI are *genetically robust* [62] (Box 1). Genetically robust genotypes are better able to maintain their phenotypic traits in the face of spontaneous mutations, and thus experience a ‘buffer’ to the effects of otherwise deleterious mutations. A genetically robust population is expected to contain extant genotypes or those that are small (e.g., single) genetic steps away from standing variants that harbor a greater diversity of neutral or nearly neutral mutational options, which may be useful for adaptation when encountering other environments. Because benefits of genetic robustness are offset by a generation (the offspring of genetically robust parents will have higher fitness than the offspring of genetically non-robust parents, but the parents themselves may have identical fitness) [64], genetic robustness may arise as a correlated consequence of another trait, such as recombination [22,65] or, in cells, well-connected metabolic networks [66]. 

In Φ6, *complementation* between different coinfecting variants (Box 1) can act as an environmentally determined mechanism that beneficially masks the fitness effects of deleterious mutations in trans. During single infections, on the other hand, the harmful effects of deleterious mutations are expressed. Frequent coinfection may thus *relax* selection for the maintenance of genetic robustness, whereas mechanisms underlying robustness should persist when single infection is more common. This hypothesis was supported by two lines of evidence. First, although populations of Φ6 evolved at a low MOI had higher genetic diversity than those evolved at a high MOI [34], the mean fitnesses of these populations were not significantly different [62], indicating that mutations in the low-MOI-evolved populations were, on average, more neutral for fitness. The second line of evidence derived from a *mutation accumulation* experiment (Box 1) using clones randomly isolated from the high-MOI-evolved and low-MOI-evolved populations. Compared with high-MOI-evolved clones, the low-MOI-evolved clones exhibited decreased fitness reductions and lesser variance in fitness changes following mutation accumulation. That low-MOI-evolved viruses experienced fewer conditionally deleterious effects of random mutational inputs indicated that prior evolution at a low MOI fostered the maintenance of genetic robustness [62].

The relationship between thermostability and genetic robustness further suggests that genetic robustness in Φ6 may operate through protein stability. Stable proteins are more likely to maintain their form and function in the face of both external thermal stress and internally destabilizing or deleterious mutations [67,68,69]. Moreover, the greater stability of these proteins should allow them to better tolerate mutations that are beneficial but destabilizing [67,69,70]. Genetic robustness, mediated through protein stability, may thus also improve protein *evolvability* [68,69,70] (Box 1). Further evolution of the low-MOI and high-MOI Φ6 populations in other, non-thermal environments could help illuminate whether their hypothesized genetic robustness also improves evolvability in environments beyond those containing elevated temperatures. 

## 6. Conclusions

Clearly, experimental evolution studies on Φ6 have contributed usefully to our understanding of various key topics in evolutionary biology, including the spontaneous nature and fitness effects of randomly occurring and adaptive mutations; the evolutionary consequences of coinfection, and its costs and benefits for viral adaptation; the mutations responsible for viruses to infect a narrow versus broad range of original and/or novel host species; and the roles of prior versus current environmental exposure in maintaining performance under stressful conditions such as heat shock. Beyond elucidating details of Φ6 and its cystovirus relatives, these studies show the use of Φ6 as a vital model for understanding the evolution of vastly different virus systems due to its traits that remain unique among known phages. The combination of a multi-partite dsRNA genome, the ability to undergo segment reassortment, and the lipid outer membrane found in Φ6 makes the virus an attractive non-pathogenic model for many viruses of disease importance, such as the Reoviridae, influenza viruses, and SARS-CoV-2. 

However, many unanswered questions still remain on the topics discussed in this review. We conclude with a select list of questions for further study.

(1) What specific mutations contribute to the evolution of cheating strategies and improved fitness only during coinfection? It remains unclear whether the evolution of cheating in Φ6 can be explained with as few as one mutation on a single RNA segment, or with multiple mutations, such as those acting via epistasis across segments.
(2) What is the frequency and degree of cost-free mutations for generalism (host-range breadth) in Φ6? Although the balance of evidence suggests that cost-free generalism in Φ6 persists in spatially heterogeneous and temporally alternating host environments, relatively few studies have measured the fitness of individual genotypes or populations in both native and novel host environments. This makes it more difficult to determine the presence and degree of fitness trade-offs. Correlated effects of mutations that primarily improve fitness on the native host, for example, could explain the tendency of Φ6 to evolve cost-free generalism.
(3) How do genotype and phenotype (fitness) change when Φ6 evolves solely on a novel host? The virology literature presents relatively few examples of point mutations that lead either to a reduction in host range or to complete specialization on a new host. There is also little evidence on how such substitutions might impact the fitness effects of additional mutations during the emergence process. This would seem an important next step in understanding the adaptation of RNA viruses after their initial emergence on a new host, and models such as Φ6 would be valuable.
(4) How does evolution under frequent versus rare opportunities for coinfection alter the broader genomic properties of Φ6, such as its ability to withstand deleterious mutations (genetic robustness) and its survival in novel environments? Evidence suggests that viruses evolved at a low MOI are putatively genetically robust and experience advantages both when infecting novel hosts and under thermal stress. However, the mechanisms that permit these advantages may not be universal. For example, low-MOI-evolved viruses did not experience enhanced survival under UV irradiation stress [63], suggesting that studies that compare low-MOI-evolved and high-MOI-evolved Φ6 populations across a wider variety of stressful conditions are warranted.
(5) Does genetic robustness, hypothesized to occur in viruses previously evolved in low-MOI conditions, improve evolvability in other, non-thermal environments? Given the particular importance of RNA viruses in disease emergence, a relationship between genetic robustness and traits such as infectivity on novel hosts should leverage tractable models such as Φ6.

## References

[1] Elena S.F., Lenski R.E. (2003). Evolution experiments with microorganisms: The dynamics and genetic bases of adaptation. Nat. Rev. Genet..

[2] Mindich L. (1988). Bacteriophage Φ6: A unique virus having a lipid-containing membrane and a genome composed of three dsRNA segments. Adv. Virus Res..

[3] Poranen M.M., Mäntynen S. (2017). ICTV virus taxonomy profile: *Cystoviridae*. J. Gen. Virol..

[4] McDonald S.M., Nelson M.I., Turner P.E., Patton J.T. (2016). Reassortment in segmented RNA viruses: Mechanisms and outcomes. Nat. Rev. Microbiol..

[5] Burch C.L., Chao L. (2004). Epistasis and its relationship to canalization in the RNA virus phi 6. Genetics.

[6] Bono L.M., Gensel C.L., Pfennig D.W., Burch C.L. (2012). Competition and the origins of novelty: Experimental evolution of niche-width expansion in a virus. Biol. Lett..

[7] Singhal S., Turner P.E. (2021). Effects of historical coinfection on host shift abilities of exploitative and competitive viruses. Evolution.

[8] Butcher S.J., Dokland T., Ojala P.M., Bamford D.H., Fuller S.D. (1997). Intermediates in the assembly pathway of the double-stranded RNA virus Φ6. EMBO J..

[9] Poranen M.M., Tuma R. (2004). Self-assembly of double-stranded RNA bacteriophages. Vir. Res..

[10] Burch C.L., Guyader S., Samarov D., Shen H. (2007). Experimental estimate of the abundance and effects of nearly neutral mutations in the RNA virus Φ6. Genetics.

[11] Muller H.J. (1964). The relation of recombination to mutational advance. Mutat. Res..

[12] Maynard Smith J. (1978). The Evolution of Sex.

[13] Chao L. (1990). Fitness of RNA Virus Decreased by Muller’s Ratchet. Nature.

[14] Burch C.L., Chao L. (2000). Evolvability of an RNA virus is determined by its mutational neighbourhood. Nature.

[15] Burch C.L., Chao L. (1999). Evolution by Small Steps and Rugged Landscapes in the RNA Virus Φ6. Genetics.

[16] Chao L., Tran T.T., Tran T.T. (1997). The advantage of sex in the RNA virus Φ6. Genetics.

[17] Fisher R. (1930). The Genetical Theory of Natural Selection.

[18] Moradigaravand D., Kouyos R., Hinkley T., Haddad M., Petropoulos C.J., Engelstädter J., Bonhoeffer S. (2014). Recombination accelerates adaptation on a large-scale empirical fitness landscape in HIV-1. PLoS Genet..

[19] Chao L., Tran T., Matthews C. (1992). Muller’s Ratchet and the Advantage of Sex in the RNA Virus Φ6. Evolution.

[20] Corbett-Detig R.B., Zhou J., Clark A.G., Harlt D.L., Ayroles J.F. (2013). Genetic incompatibilities are widespread within species. Nature.

[21] Kvitek D.J., Sherlock G. (2013). Reciprocal sign epistasis between frequently experimentally evolved adaptive mutations causes a rugged fitness landscape. PLoS Genet..

[22] Singhal S., Gomez S.M., Burch C.L. (2019). Recombination drives the evolution of mutational robustness. Curr. Opin. Syst. Biol..

[23] Turner P.E., Chao L. (1998). Sex and the evolution of intrahost competition in RNA virus Φ6. Genetics.

[24] Mindich L., Sinclair J.F., Levine D., Cohen D. (1976). Genetic studies of temperature-sensitive and nonsense mutants of bacteriophage φ6. Virology.

[25] Onodera S., Sun Y., Mindich L. (2001). Reverse genetics and recombination in φ8, a dsRNA bacteriophage. Virology.

[26] Silander O.K., Weinreich D.M., Wright K.M., O’Keefe K.J., Rang C.U., Turner P.E., Chao L. (2005). Widespread genetic exchange among terrestrial bacteriophages. Proc. Natl. Acad. Sci. USA.

[27] Gottlieb P., Alimova A. (2022). Heterologous RNA Recombination in the Cystoviruses φ6 and φ8: A Mechanism of Viral Variation and Genome Repair. Viruses.

[28] Turner P.E., Chao L. (1999). Prisoner’s dilemma in an RNA virus. Nature.

[29] Dennehy J.J., Turner P.E. (2004). Reduced fecundity is the cost of cheating in RNA virus Φ6. Proc. R. Soc. Lond. B.

[30] Turner P.E., Chao L. (2003). Escape from prisoner’s dilemma in RNA phage phi-6. Am. Nat..

[31] Huang A.S., Baltimore D. (1970). Defective viral particles and viral disease processes. Nature.

[32] Huang A.S. (1973). Defective interfering viruses. Annu. Rev. Microbiol..

[33] Horiuchi K. (1983). Co-evolution of filamentous bacteriophage and its defective interfering particles. J. Mol. Biol..

[34] Dennehy J.J., Duffy S., O’Keefe K.J., Edwards S.V., Turner P.E. (2013). Frequent coinfection reduces RNA virus population genetic diversity. J. Hered..

[35] Froissart R., Wilke C.O., Montville R., Remold S.K., Chao L., Turner P.E. (2004). Coinfection weakens selection against epistatic mutations in RNA viruses. Genetics.

[36] Simon-Loriere E., Holmes E.C. (2011). Why do RNA viruses combine?. Nat. Rev. Microbiol..

[37] Woolhouse M.E.J., Taylor L.H., Haydon D.T. (2001). Population biology of multihost pathogens. Science.

[38] Woolhouse M.E.J., Gowtage-Sequeria S. (2005). Host range and emerging and reemerging pathogens. Emerg. Infect. Dis..

[39] Turner P.E., Morales N.M., Alto B.W., Remold S.K. (2010). Role of evolved host breadth in the initial emergence of an RNA virus. Evolution.

[40] Remold S. (2012). Understanding specialism when the jack of all trades can be the master of all. Proc. R. Soc. B.

[41] Singh B.B., Ward M.P., Dhand N.K. (2023). Host characteristics and their influence on zoonosis, disease emergence and multi-host pathogenicity. One Health.

[42] Berge O., Monteil C.L., Bartoli C., Chandeysson C., Guilbaud C. (2014). A user’s guide to a data base of the diversity of *Pseudomonas syringae* and its application to classifying strains in this phylogenetic complex. PLoS ONE.

[43] Gomila M., Busquets A., Mulet M., Garcia-Valdés E., Lalucat J. (2017). Classification of taxonomic status within the *Pseudomonas syringae* species group based on phylogenomic analysis. Front. Microbiol..

[44] Duffy S., Turner P.E., Burch C.L. (2006). Pleiotropic costs of niche expansion in the RNA bacteriophage phi6. Genetics.

[45] Ferris M.T., Joyce P., Burch C.L. (2007). High frequency of mutations that expand the host range of an RNA virus. Genetics.

[46] Zhao L., Duffy S. (2019). Gauging genetic diversity of generalists: A test of genetic and ecological generalism with RNA virus experimental evolution. Virus Evol..

[47] Zhao L., Seth-Pasricha M., Stemate D., Crespo-Bellido A., Gagnon J., Draghi J., Duffy S. (2019). Existing host range mutations constrain further emergence of RNA viruses. J. Virol..

[48] Dennehy J.J., Friedenberg N.A., Holt R.D., Turner P.E. (2006). Viral ecology and the maintenance of novel host use. Am. Nat..

[49] Duffy S., Burch C.L., Turner P.E. (2007). Evolution of host specificity drives reproductive isolation among RNA viruses. Evolution.

[50] Ford B.E., Sun B., Carpino J., Chapler E.S., Ching J., Choi Y., Jhun K., Kim J.D., Lallos G.G., Morgenstern R. (2014). Frequency and fitness consequences of bacteriophage φ6 host range mutations. PLoS ONE.

[51] Bono L.M., Gensel C.L., Pfennig D.W., Burch C.L. (2015). Evolutionary rescue and the coexistence of generalist and specialist competitors. Proc. R. Soc. B.

[52] Bono L.M., Smith L.B., Pfennig D.W., Burch C.L. (2016). The emergence of performance trade-offs during local adaptation: Insights from experimental evolution. Mol. Ecol..

[53] Bedhomme S., Hillung J., Elena S.F. (2015). Emerging viruses: Why they are not jacks of all trades?. Curr. Opin. Virol..

[54] McBride R.C., Ogbunugafor C.B., Turner P.E. (2008). Robustness promotes evolvability of thermotolerance in an RNA virus. BMC Evol. Biol..

[55] Dessau M., Goldhill D., McBride R.C., Turner P.E., Modis Y. (2012). Selective pressure causes an RNA virus to trade reproductive fitness for increased structural and thermal stability of a viral enzyme. PLoS Genet..

[56] Singhal S., Leon Guerrero C.M., Whang S.G., McClure E.M., Busch H.G., Kerr B. (2017). Adaptations of an RNA virus to increasing thermal stress. PLoS ONE.

[57] Goldhill D., Lee A., Williams E.S.C.P., Turner P.E. (2014). Evolvability and robustness in populations of RNA virus φ6. Front. Microbiol..

[58] Mindich L., Lehman J. (1979). Cell wall lysin as a component of the bacteriophage Φ6 virion. J. Virol..

[59] Olkkonen V.M., Ojala P., Bamford D.H. (1991). Generation of infectious nucleocapsids by in vitro assembly of the shell protein onto the polymerase complex of the dsRNA bacteriophage Φ6. J. Mol. Biol..

[60] Somero G.N. (1995). Proteins and temperature. Annu. Rev. Physiol..

[61] DePristo M.A., Weinreich D.M., Hartl D.L. (2005). Missense meanderings in sequence space: A biophysical view of protein evolution. Nat. Rev. Gen..

[62] Montville R., Froissart R., Remold S.K., Tenaillon O., Turner P.E. (2005). Evolution of mutational robustness in an RNA virus. PLoS Biol..

[63] Ogbunugafor C.B., McBride R.C., Turner P.E. (2009). Predicting virus evolution: The relationship between genetic robustness and evolvability of thermotolerance. Cold Spring Harb. Symp..

[64] Wagner A. (2005). Robustness and Evolvability in Living Systems.

[65] Azevedo R.B.R., Lohaus R., Srinivasan S., Dang K.K., Burch C.L. (2006). Sexual reproduction selects for robustness and negative epistasis in artificial gene networks. Nature.

[66] Siegal M.L., Bergman A. (2002). Waddington’s canalization revisited: Developmental stability and evolution. Proc. Natl. Acad. Sci. USA.

[67] Ancel L.W., Fontana W. (2000). Plasticity, evolvability, and modularity in RNA. J. Exp. Zool..

[68] Bloom J.D., Labthavikul S.T., Otey C.R., Arnold F.H. (2006). Protein stability promotes evolvability. Proc. Natl. Acad. Sci. USA.

[69] Gong L.I., Suchard M.A., Bloom J.D. (2013). Stability-mediated epistasis constrains the evolution of an influenza protein. eLife.

[70] Tokuriki N., Tawfik D.S. (2009). Stability effects of mutations and protein evolvability. Curr. Opin. Struct. Biol..

